# Microbes mediated comprehensive carbon sequestration for negative emissions in the ocean

**DOI:** 10.1093/nsr/nwaa171

**Published:** 2020-07-28

**Authors:** Nianzhi Jiao, Jihua Liu, Fanglue Jiao, Quanrui Chen, Xiaoxue Wang

**Affiliations:** State Key Laboratory of Marine Environmental Science and College of Ocean and Earth Sciences, Xiamen University, China; Joint Lab for Ocean Research and Education (LORE) of Dalhousie University, Canada, and Shandong University and Xiamen University, China; Joint Lab for Ocean Research and Education (LORE) of Dalhousie University, Canada, and Shandong University and Xiamen University, China; Institute of Marine Science and Technology, Shandong University, China; Joint Lab for Ocean Research and Education (LORE) of Dalhousie University, Canada, and Shandong University and Xiamen University, China; State Key Laboratory of Marine Environmental Science and College of Ocean and Earth Sciences, Xiamen University, China; Joint Lab for Ocean Research and Education (LORE) of Dalhousie University, Canada, and Shandong University and Xiamen University, China; South China Sea Institute of Oceanology, Chinese Academy of Sciences, China

The crisis of the COVID-19 pandemic is warning of a more profound crisis—climate change. Since the Industrial Revolution, anthropogenic activities, such as the burning of fossil fuels and deforestation, have led to a significant increase in atmospheric CO_2_ concentration, exacerbating climate change and causing ecosystem imbalances, abrupt ecosystem successions and serious ecological disasters, which will finally threaten the sustainable development of human society.

What we can do to mitigate climate change is to minimize greenhouse-gas emissions in daily life and economic practice. The effects of such countermeasures happened to have been tested out by the COVID-19 pandemic event, during which an abrupt drop in CO_2_ emissions equivalent to 17% of the total for 2019 was recorded in the first four months of 2020 [1]. Significant improvements in air quality have been observed across countries, such as a 30% reduction in pollution in northeastern USA and the first sighting of the Himalayas from Indian cities for >30 years. However, such a blessing in disguise will not last for long, just as the emission fall during the 2008 financial crisis was followed by an immediate emission increase of 5% in 2009. There will very likely be a rebound in emissions post COVID-19.

While it is well understood that a feasible way to mitigate climate change at present is to reduce CO_2_ emissions to zero, it is unrealistic to achieve this goal in the short term. Under such circumstances, an alternative scheme to remove CO_2_ from the atmosphere has become urgently necessary, i.e. negative emission. Many approaches have been proposed in the literature in this regard, among which an emerging area—the marine carbon sink—is particularly promising. In fact, the ocean is the largest carbon reservoir on the planet and did play an important role in regulating climate change in the history of Earth. Ocean negative carbon emission (ONCE) has been proposed for an international program [2] towards the goal of limiting global warming to the 1.5–2°C targets of the Paris Agreement (UNFCCC).

**Figure 1.**
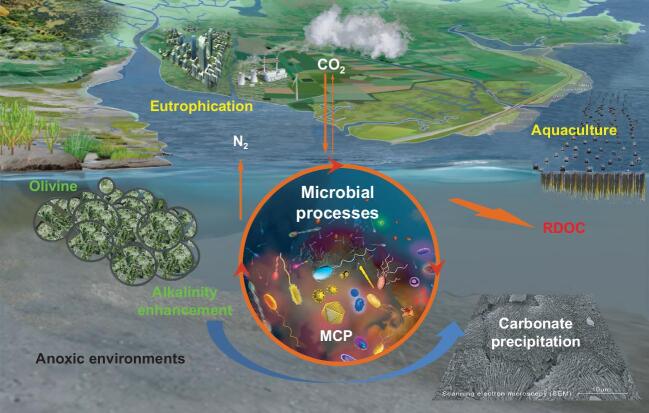
A demo of the microbe-mediated ocean carbon negative emission (ONCE) processes in anoxic environments, including the production of refractory dissolved organic carbon (RDOC), enhancement of alkalinity and precipitation of carbonates. The lower right panel is a scanning electron microscopic picture of authigenic carbonates observed in a simulated experiment.

A natural mechanism for the ocean to absorb CO_2_ from the atmosphere is the solubility pump. Along with the persistent increase in the anthropogenic emission of CO_2_ and the consequent transmission to the ocean, seawater chemistry has been changed toward acidification, consequently resulting in ecological disasters. To cope with ocean acidification, it is critical to maintain seawater alkalinity. The total alkalinity of seawater is generated by erosion/weathering processes on land and metabolic processes in the ocean. However, terrestrial runoffs also discharge a great deal of nutrients into coastal waters, causing eutrophication, algal blooms and hypoxia, jeopardizing ecosystem sustainability [3]. Recent progress in the understanding of ocean carbon sequestration is the microbial carbon pump (MCP) that transforms organic carbon from labile to refractory dissolved organic carbon (RDOC) constituting long-term carbon storage in the water column [4] (Fig. 1, lower panel, middle), intervening with the known biological pump that transports particulate organic carbon to the sediment, making it possible for ecosystem manipulations for ONCE countermeasures even in such vulnerable environments as coastal eutrophication waters and aquaculture systems [5]. Particularly in situations of eutrophication and hypoxia, microbial anaerobic respiration metabolism produces less CO_2_ and more bicarbonate coupled with N_2_ as the end product by the denitrification process, making an irreversible path from nitrate to N_2_ that escapes from the water into the air and thus alleviates eutrophication while increasing the seawater alkalinity [6]. As seen from our preliminary experiments simulating the sediment environment of massive aquaculture, MCP-mediated biogeochemical processes could sequester carbon in the forms of RDOC [4], alkalinity [6] and authigenic carbonates (Fig. 1), reproducing the scenarios of a large amount of carbon storage in the history of Earth [7]. Even in the current oceans, RDOC is a huge carbon reservoir equivalent in total amount to the carbon inventory of CO_2_ in the atmosphere [4] and authigenic carbonates account for ≥10% of global carbonates [8]. Rock records reveal that Precambrian stromatolites formed as a consequence of the trapping and precipitation of calcium carbonate by microbial metabolisms. The metabolic activities of anaerobic methanotrophic archaea and sulfate-reducing bacteria can enhance the concentration of bicarbonate, a major component of total alkalinity. The increase in alkalinity then promotes the combination of microbial mats/biofilm with Ca^2+^ ions to induce carbonate deposition. Recent studies have shown that the microbial extracellular polymeric substances facilitate carbonate precipitation via serving as nucleation sites for mineral growth [9]. The main components of the mineral precipitates form an organic–inorganic hybrid film on a solid surface and it is composed of biofilm and calcite (Mg_0.064_Ca_0.936_)(CO_3_) (Fig. 1, lower panel, right). The same is true for the formation of the mineral crystals of aragonite and dolomite. These authigenic carbonates gradually increase and become mineral rocks with microbial attachment and cementation processes.

Alkalization can also be achieved by accelerating the dissolution of the silicate-containing minerals such as olivine, which is a magnesium silicate compound (Mg_2_SiO_4_) abundant in Earth's crust. Olivine dissolution is part of the natural silicate weathering process, responsible for naturally sequestering CO_2_ at a rate of ∼0.5 billion tons per year [10]. The chemical-reaction equation (Mg_2_SiO_4_ + 4CO_2_ + 4H_2_O  ⇒2Mg^2+^ + 4HCO_3_^−^ + H_4_SiO_4_) suggests that 4 mol of CO_2_ can be sequestered by 1 mol of olivine, equivalent to 1.25 t of CO_2_ per ton of olivine. The efficiency of dissolution is critical for this approach in application in terms of cost, which is limited by olivine grain sizes and environmental conditions. Our preliminary experimental results showed that olivine mineral dissolution efficiency can be enhanced by the microbial processes that adjust the chemical-reaction balance, making the olivine ONCE approach practically promising (Fig. 1, lower panel).

Taken together, the MCP-mediated production of RDOC, enhancement of alkalinity and precipitation of carbonates in hypoxic coastal eutrophication waters and massive aquaculture areas would be a promising ONCE approach to kill two birds with one stone: increasing carbon sinks while improving the environment (Fig. 1).
